# Colorectal Cancer in Brazil: Regional Disparities and Temporal Trends in Diagnosis and Treatment, 2013–2024

**DOI:** 10.3390/diseases14020040

**Published:** 2026-01-26

**Authors:** Luiz Vinicius de Alcantara Sousa, Jean Henri Maselli-Schoueri, Laércio da Silva Paiva, Bianca Alves Vieira Bianco

**Affiliations:** 1 Laboratory of Epidemiology and Data Analysis, FMABC University Center, Santo André 09060-870, SP, Brazil; laercio.paiva@fmabc.net; 2 Princess Margaret Cancer Centre, Division of Medical Oncology and Hematology, Department of Medicine, Melanoma and Skin Cancer, University of Toronto, 610 University Avenue, Toronto, ON M5G 2M9, Canada; jean.schoueri@gmail.com; 3 Discipline of Sexual and Reproductive Health and Population Genetics, Department of Public Health, FMABC University Center, Santo André 09060-870, SP, Brazil; bianca.bianco@hotmail.com

**Keywords:** colorectal cancer, regional disparities, health inequalities, public health

## Abstract

Background/Objectives: Colorectal cancer (CRC) is a major public health challenge in Brazil, characterized by marked regional disparities. Although national legislation mandates that treatment begin within 60 days after diagnosis, compliance remains inconsistent, particularly within the Unified Health System (SUS). This study aimed to analyze the time to treatment initiation for colon (C18) and rectal (C20) cancer in Brazil from 2013 to 2024, assessing regional inequalities, temporal trends, and factors associated with treatment delays. Methods: We conducted an ecological study using secondary data from the Ministry of Health’s PAINEL-Oncologia platform, which integrates information from SIA/SUS, SIH/SUS, and SISCAN. Records of patients diagnosed with colon and rectal cancer (ICD-10 C18–C20) were evaluated. Temporal trends were analyzed using Joinpoint regression, and factors associated with delayed treatment initiation (>60 days) were identified through multiple logistic regression models. Results: Persistent discrepancies were observed between diagnostic and treatment trends from 2013 to 2024, with the Annual Percent Change (APC) for diagnosis exceeding that for treatment, particularly among adults aged 55–69 years. The Southeast and South regions accounted for over 70% of all diagnosed cases, starkly contrasting with the less than 25% in the North and Northeast. More than 50% of patients across all clinical stages initiated treatment after the legally mandated 60-day period. Women with rectal cancer had a 28% higher risk (RR = 1.28) of being diagnosed at stage IV. Chemotherapy was the predominant initial therapeutic modality, while the need for combined chemo-radiotherapy was associated with markedly elevated risk ratios for delay (e.g., RR = 26.53 for stage IV rectal cancer). Treatment initiation delays (>60 days) were significantly associated with residence in the North/Northeast regions, female sex (for rectal cancer), advanced-stage disease, and complex therapeutic regimens. Conclusions: The study demonstrates persistent regional inequalities and highlights a substantial mismatch between diagnostic capacity and therapeutic availability in Brazil. These gaps contribute to treatment delays and reinforce the need to strengthen and expand oncological care networks to ensure equitable access and improve outcomes, particularly in underserved regions.

## 1. Introduction

Colorectal cancer (CRC), which includes malignant neoplasms of the colon (C18) and rectum (C20), is a major global health challenge. It is currently the third most common cancer and the second leading cause of cancer-related mortality worldwide, with nearly 1.9 million new cases and over 900,000 deaths in 2020 [1]. Projections indicate that its global burden will continue to rise, especially in low- and middle-income countries undergoing rapid demographic and nutritional transitions [2].

In Brazil, CRC has become one of the most incident malignant neoplasms. For the 2024–2025 period, the National Cancer Institute (INCA) estimates approximately 45,000 new cases annually, ranking CRC as the second most frequent cancer in men and women [3]. However, this burden is not equally distributed: the South and Southeast show the highest incidence rates, whereas the North and Northeast continue to report lower, but increasing, rates, reflecting pronounced regional inequalities in socioeconomic development, healthcare infrastructure, and access to diagnostic services [4,5,6,7,8].

Timely diagnosis and the initiation of treatment are crucial determinants of CRC outcomes. Early-stage cases have markedly better survival than advanced disease [9], yet delays remain common in regions with limited access to colonoscopy, imaging, and specialized oncology services [10]. In response, Brazil enacted Law No. 12.732/2012, establishing a maximum interval of 60 days between diagnosis and the start of oncological treatment within the Unified Health System (SUS). Despite this regulatory milestone, studies indicate persistent noncompliance, especially in resource-constrained settings [11,12,13,14,15,16,17], contributing to avoidable morbidity, mortality, and regional disparities [7,18].

Although previous research has examined specific components of CRC care—including mortality trends, treatment delays, and diagnostic gaps—comprehensive analyses integrating temporal trends, regional inequalities, and factors associated with delayed treatment initiation remain limited in the Brazilian context, particularly when considering the disruptions in cancer diagnosis and treatment observed during the COVID-19 pandemic [19,20,21,22]. Furthermore, the availability of new nationwide data through the Ministry of Health’s PAINEL-Oncologia platform provides an opportunity to explore these dynamics in greater depth [23,24,25].

Given these gaps, the present study aims to analyze the time to treatment initiation for colon (C18) and rectal (C20) cancer in Brazil from 2013 to 2024, examining temporal trends, regional inequities, and sociodemographic and clinical factors associated with delays. The study provides evidence essential for improving oncological care pathways and addressing persistent inequalities in access to diagnosis and treatment across Brazilian regions.

## 2. Materials and Methods

### 2.1. Study Design

This was an ecological study based on secondary data obtained from the Oncological Treatment Monitoring Panel (PAINEL-Oncologia), a public platform maintained by the Brazilian Ministry of Health. The study period covered the years 2013 to 2024.

### 2.2. Data Source

Data were extracted from PAINEL-Oncologia, which compiles information from three national health information systems:

(a) the SUS Outpatient Information System (SIA/SUS);
(b) the SUS Hospital Information System (SIH/SUS); and
(c) the Cancer Information System (SISCAN).

The extracted dataset included variables related to:

- geographic region and federative unit of residence, diagnosis, and treatment;
- demographic characteristics (sex, age, age group);
- cancer type according to ICD-10;
- clinical staging at diagnosis;
- therapeutic modality (surgery, chemotherapy, radiotherapy);
- and time to treatment initiation (days).

All data used were fully anonymized and publicly accessible.

### 2.3. Study Population

We included all patients diagnosed with colon or rectal cancer (ICD-10: C18–C20) between 2013 and 2024 within the Brazilian Unified Health System (SUS). Malignant neoplasms of the rectosigmoid junction (C19) were excluded due to their lower frequency and distinct clinical behavior.

Inclusion Criteria:

(a) confirmed diagnosis of colon or rectal cancer;
(b) complete information on diagnosis, therapeutic modality, and time to treatment initiation.

Exclusion criteria:

(a) incomplete or inconsistent records (e.g., missing dates of diagnosis or treatment);
(b) cases in which treatment initiation occurred outside the study period.

### 2.4. Study Variables

Dependent variable:
  Time to treatment initiation, categorized as:
    (1) ≤30 days;
    (2) 31–60 days;
    (3) >60 days.
Independent variables:
  Demographic characteristics:
    Sex (male, female); age (stratified into 5-year groups: 40–44, 45–49, 50–54, 55–59, 60–64, 65–69, 70–74, 75–79, ≥80 years); region and federative unit of residence.
  Clinical characteristics:
    Clinical staging (carcinoma in situ, I, II, III, IV) according to PAINEL-Oncologia/INCA–DATASUS; therapeutic modality (surgery, chemotherapy, radiotherapy); cancer subtype (colon, rectal).
  Regional characteristics:
    Federative unit of diagnosis; federative unit of treatment.

### 2.5. Statistical Analysis

Descriptive analyses were performed using absolute and relative frequencies of demographic, clinical, and regional variables. Treatment rates were age-standardized using the world standard population (Segi–Doll) and expressed as age-standardized rates (ASR) per 100,000 inhabitants.

Temporal trends (2013–2024) were evaluated using Joinpoint regression (Joinpoint Regression Program, version 4.9.1.0), which estimates inflection points and computes the Annual Percent Change (APC) with 95% confidence intervals.

Multiple logistic regression models were used to identify factors independently associated with delayed treatment initiation (>60 days), adjusting for age, sex, region of residence, clinical stage, and treatment modality. Statistical analyses were performed using Stata (version 18.0; StataCorp LLC, College Station, TX, USA), with statistical significance set at *p* < 0.05.

To describe the relative distribution of cases across categories of key variables (sex, treatment delay, treatment modality, and geographic region) within each cancer stage, proportion ratios (PRs) were calculated. The PR represents the relationship between the proportion of cases in a given category and the proportion observed in the corresponding reference category within the same variable and clinical stage. For example, in the analysis of sex distribution among patients with stage IV rectal cancer, the PR for women corresponds to the proportion of women at this stage relative to the proportion of men. Reference categories were defined as follows: male for sex, treatment delay ≤ 30 days, chemotherapy as the treatment modality, and the North region. These measures allow a direct comparison of how cases are distributed across categories within each clinical stage.

### 2.6. Ethical Considerations

This study did not require approval by a Research Ethics Committee because it relied exclusively on publicly available, anonymized secondary data from PAINEL-Oncologia. According to Resolution No. 510/2016 of the Brazilian National Health Council, research using public and non-identifiable information is exempt from ethical review.

### 2.7. Use of Generative Artificial Intelligence

Generative artificial intelligence (GenAI) was used solely for language refinement, organization, and formatting of the manuscript text. GenAI was not used for data collection, statistical analysis, interpretation of results, or generation of datasets, figures, or study design.

## 3. Results

**Figure 1:**
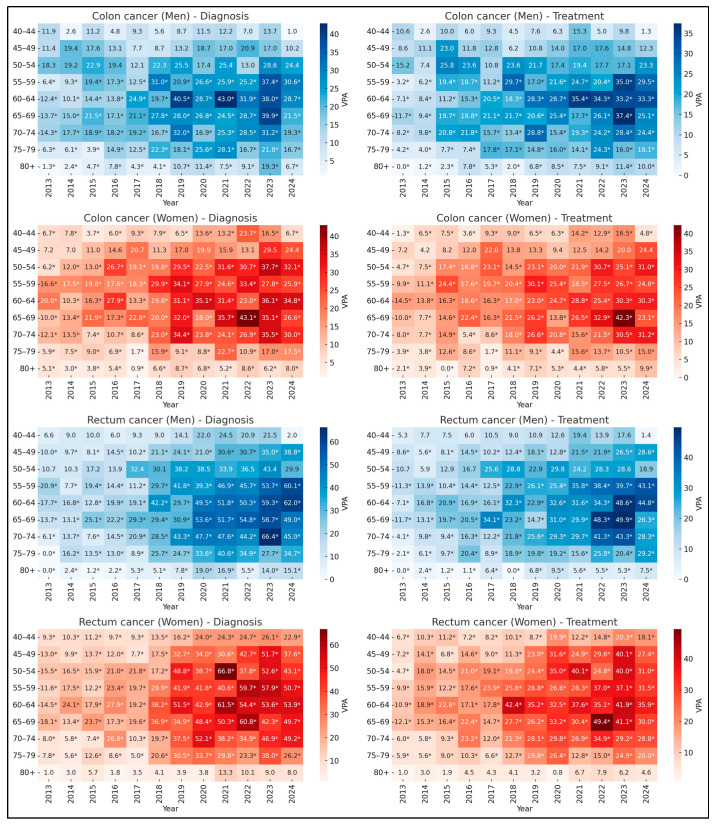
Annual Percent Change (APC) in the diagnosis and treatment of colon (C18) and rectal (C20) cancer, by age group and sex, Brazil, 2013-2024. Legend: Heatmaps depict the Annual Percent Change (APC) for diagnosis (**left panels**) and treatment (**right panels**). The APC represents the average annual percent change in age-adjusted rates. Darker shades indicate higher APC values. Each cell displays the APC for the corresponding year and age group. An asterisk (*) denotes a statistically significant temporal trend (*p* < 0.05) according to Prais-Winsten regression.

Comparative analyses of temporal trends from 2013 to 2024 revealed consistent discrepancies between diagnosis and treatment rates for colorectal cancer (CRC) in Brazil. Joinpoint regression analysis demonstrated that annual increases in diagnostic rates generally outpaced growth in treatment initiation, particularly among middle-aged adults (55–69 years). As illustrated in Figure 1, which presents heatmaps of the Annual Percent Change (APC) in age-adjusted rates for colon (C18) and rectal (C20) cancer stratified by sex and age group, darker colors indicate higher APC values for each year. For example, among men aged 55–64 years with colon cancer, the APC for diagnosis (β = 2.17–2.73; *p* < 0.001) exceeded that for treatment (β = 1.95–2.73; *p* < 0.001). A similar pattern is visually evident for rectal cancer, notably among men aged 60–74 years and women aged 55–69 years, where diagnostic cells are consistently darker than their treatment counterparts. In contrast, trends remained stable in younger adults (40–49 years) and showed stabilization or decline in older age groups (≥75 years). These findings suggest that the expansion of diagnostic capacity has not been proportionally matched by therapeutic coverage, pointing to systemic bottlenecks in care continuity.

The geographical distribution of colorectal cancer (CRC) diagnoses in Brazil (2013–2024) showed a pronounced concentration in the more developed Southeast and South regions, which together comprised over 70% of all colon and rectal cancer cases (Table 1). The Southeast alone accounted for nearly half of the diagnoses (47.87% of colon, 48.12% of rectal cancers), followed by the South with approximately one-quarter. In contrast, the North and Northeast together represented less than 25% of cases, with the North contributing the smallest share (≤5% for both cancer types). This distribution underscores profound regional inequalities, likely influenced by disparities in healthcare infrastructure, population aging, and access to diagnostic services, which may contribute to both lower incidence estimates and underdiagnosis in less developed regions.

Regional patterns in treatment initiation mirrored those observed for diagnoses, with the highest Annual Percent Changes (APCs) concentrated in the Southeast and South (Table 2). In the Southeast, APCs peaked among adults aged 60–64, reaching 90.28 for men with colon cancer. The South showed even higher growth in specific groups, such as men aged 65–69 with colon cancer (APC = 138.21). In stark contrast, the North exhibited the lowest increases, with APCs below 5.0 in older age groups (e.g., 4.26 for men aged ≥80 with colon cancer). This gradient reinforces profound regional disparities in therapeutic access.

Analysis of rectal cancer staging revealed notable sex and regional disparities. Men represented the majority of cases across all stages (53.9–56.2%). Using men as the reference group, women showed higher risk ratios (RR) for advanced-stage diagnosis, reaching 1.28 for stage IV, suggesting a greater propensity for late-stage diagnosis among women. Delays in treatment initiation were common, with over half of patients beginning therapy after 60 days across all stages. Early initiation (≤30 days) was associated with a significantly reduced risk (RR 0.26–0.39). Chemotherapy was the predominant treatment modality, especially in stage IV (77.3% of cases). Using chemotherapy as the reference, radiotherapy and combined chemo-radiotherapy were less frequent but were associated with elevated RRs (reaching 3.91 for radiotherapy and 26.53 for combined therapy in stage IV), indicating that these modalities were more commonly used in complex, advanced presentations. Regionally, with the North as the reference, risk ratios for other regions were consistently below 1.00, suggesting a proportionally greater burden of late-stage disease in the North (Table 3).

**Figure A1:**
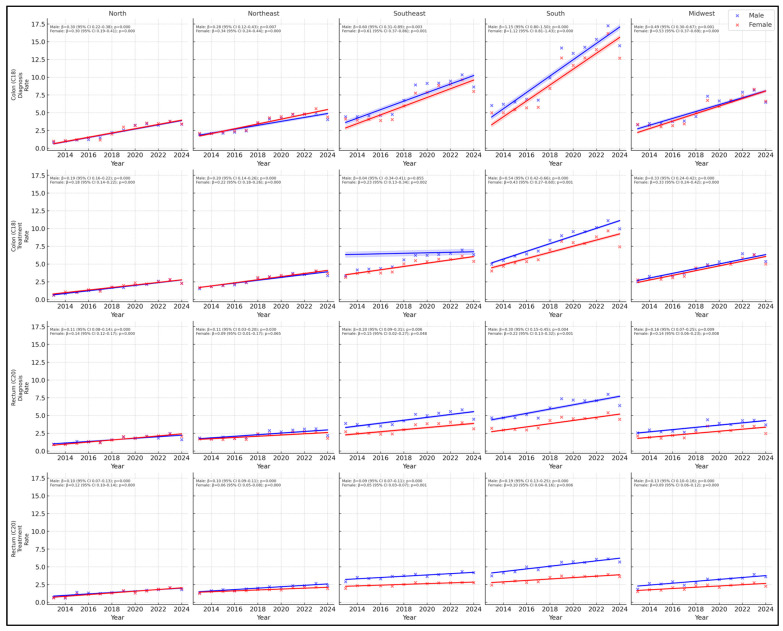
Temporal trends in diagnosis and treatment of colon (C18) and rectum (C20) cancer in Brazil by region and sex, 2013–2024.

For colon cancer, the distribution of clinical stages showed minimal variation by sex, with nearly equal proportions across stages. Using men as the reference group, risk ratios (RR) for women were close to 1.00, indicating no significant sex-based difference in stage distribution. Treatment delays were prevalent, with over half of patients initiating therapy after 60 days regardless of stage. Using early treatment (≤30 days) as the reference, longer delays were associated with elevated risk (e.g., RR > 60 days = 2.11–3.76). Chemotherapy dominated as the primary treatment modality (≥97% of cases). Compared with chemotherapy as the reference modality, radiotherapy (<3% of cases) was associated with markedly reduced RRs (e.g., 0.01 in stage IV), reflecting its limited and selective use in complex presentations. Combined chemo-radiotherapy was exceedingly rare. Regionally, with the North as the reference, risk ratios for other regions remained below 1.00, suggesting a proportionally greater burden of advanced disease in the North (Table 4). Temporal trends by sex and Brazilian region are presented in Appendix A (Figure A1).

Analysis of colon cancer treatment patterns reaffirmed previous findings: sex distribution in treatment access was balanced (RR for females 0.98–1.10), and delays exceeding 60 days were common across stages (~50% of cases). Chemotherapy remained the cornerstone of treatment (>97% of cases), while radiotherapy (≤3%) was associated with elevated risk ratios, indicative of its role in complex care. Regionally, treatment provision was heavily concentrated, with the Southeast and South accounting for nearly three-quarters of stage IV treatments (47.1% and 26.9%, respectively). In contrast, the North and Midwest together contributed less than 10%. Risk ratios for treatment access were markedly higher in the Southeast (RR up to 20.68) and South (RR 11.78) relative to the North, underscoring severe geographic inequities in therapeutic resource allocation (Table 5).

Treatment patterns for rectal cancer revealed a consistent male predominance across stages (54.0% in carcinoma in situ to 50.7% in stage IV), with risk ratios (RR) for women remaining near 1.00. Treatment delays exceeding 60 days were prevalent, affecting over half of patients in all stages. Early initiation (≤30 days) was associated with substantially reduced risk (RR 0.29–0.41). Chemotherapy was the primary modality, especially in stage IV (80.1%), while radiotherapy (19.9% in stage IV, RR = 0.25) and combined chemo-radiotherapy (RR up to 27.24) were linked to more complex presentations. Geographically, treatment access was heavily skewed, with the Southeast and South accounting for nearly three-quarters of stage IV treatments (49.3% and 24.7%, respectively). Risk ratios for treatment were markedly elevated in these regions relative to the North (RR up to 18.14 and 9.09), confirming pronounced geographic inequities in service provision (Table 6).

## 4. Discussion

This study outlines a complex and heterogeneous landscape of colorectal cancer (CRC) treatment within Brazils Unified Health System (SUS) from 2013 to 2024. As an ecological analysis based on population level administrative data, the associations reported here reflect systemic patterns and should not be interpreted as establishing causal relationships at the individual patient level. The observed mismatch between the growth in diagnostic activity and therapeutic coverage, most apparent among adults aged 55 to 69 years, points to structural health system constraints that transcend purely organizational factors [26,27].

A pronounced regional concentration of diagnosed cases was evident, with nearly 70% occurring in the Southeast and South, compared to only 24% in the North and Northeast. This imbalance underscores enduring socioeconomic and healthcare inequities, shaped by historical underinvestment in health infrastructure, differential exposure to risk factors, and unequal access to specialized oncology services across regions [8,28,29]. A critical dimension of this disparity is the unequal distribution of specialized human resources. Regions such as the North and Northeast face significant shortages of gastroenterologists, oncological surgeons, clinical oncologists, and radiation oncologists, which directly constrains both diagnostic capacity (e.g., colonoscopy availability) and the timely initiation of multidisciplinary treatment. These patterns are consistent with Brazil’s ongoing epidemiological transition, wherein more developed regions report higher cancer incidence linked to aging populations and lifestyle-related risks [30].

Despite the legal mandate established by Law No. 12.732/2012, which requires treatment initiation within 60 days of diagnosis, more than half of patients across all clinical stages exceeded this timeframe. While these findings highlight a systemic shortcoming, they must be interpreted cautiously, given the known limitations of administrative datasets, which may not fully capture the nuances of individual care pathways [31,32].

Caution is also warranted when interpreting the risk ratios (RRs) derived from regional and treatment modality analyses. Extremely high RR values, particularly in comparisons where the reference category had low case counts such as the North region, likely reflect data sparsity and structural imbalances in case distribution. Such estimates should be regarded as indicators of substantial disparity rather than precise measures of effect size.

The association between multimodal therapy and longer waiting times suggests that patients with more complex care needs face additional systemic barriers. These may include the necessity for multidisciplinary assessment, specialized surgical scheduling, and coordination across multiple service points [33]. Within the SUS, where demand often exceeds the availability of specialized resources, such coordination challenges can create significant bottlenecks in the care pathway [34].

Older adults and patients diagnosed with metastatic disease were more likely to experience treatment delays, reflecting both clinical complexity and systemic inefficiencies [35,36]. Older patients frequently present with comorbidities that require comprehensive pre therapeutic evaluation, while metastatic cases necessitate detailed staging and multidisciplinary planning [37]. Importantly, these delays may mask deeper regional inequities in access to diagnostic technologies, specialist professionals, and oncology reference centers [38].

It should be noted that although treatment delays are widely regarded as adversely affecting cancer outcomes, this study did not assess survival or other clinical endpoints. Consequently, our conclusions focus primarily on health system performance and access to care, rather than on direct patient outcomes. Future research incorporating clinical and survival data is needed to better quantify the prognostic impact of treatment delays in the Brazilian context.

The COVID-19 pandemic substantially disrupted cancer care delivery in Brazil, with documented reductions in diagnostic procedures, delays in treatment initiation, and increased cancer-related mortality during 2020 and 2021 [32,33,34,35,36]. These disruptions likely aggravated pre-existing regional inequities and may have influenced the temporal trends observed in the latter phase of the study period. Although our analytical models did not explicitly adjust for pandemic-related effects, this contextual factor should be considered when interpreting trends from 2020 onward.

### 4.1. Study Limitations

Several limitations should be acknowledged. The exclusive reliance on SUS administrative data may result in underreporting, particularly in regions with lower public health coverage, and variability in data quality across states could affect trend analyses. The ecological design precludes causal inference, and the lack of individual level data on comorbidities, socioeconomic status, and tumor characteristics limits the ability to adjust for potential confounders. Additionally, elevated RRs in some regional comparisons may stem from population composition differences or selection biases not captured in the dataset.

### 4.2. Implications and Future Directions

The regional disparities documented here mirror broader socioeconomic divides in Brazil [37]. Lower diagnostic proportions in the North and Northeast may indicate either genuinely lower incidence or substantial underdiagnosis due to limited screening coverage and restricted access to diagnostic endoscopy [38,39]. The concentration of treatments in the Southeast and South may also reflect unrecorded patient migration, which places additional logistical and financial burdens on families [40].

Addressing these inequities will require integrated strategies that expand diagnostic capacity while concurrently investing in therapeutic infrastructure, including surgical, radiotherapy, and specialized human resources. The implementation of patient navigation programs could help reduce delays, and the adoption of standardized protocols with real-time monitoring may assist in identifying and resolving systemic bottlenecks. Future studies should aim to incorporate survival analyses, integrate PAINEL Oncologia data with population-based cancer registries, and develop granular quality indicators for oncology care, particularly time to treatment metrics stratified by region and clinical stage.

## 5. Conclusions

The results of this study show that, despite advances in recent years, Brazil still faces substantial challenges in ensuring timely and equitable access to colorectal cancer treatment. The observed delays and regional disparities reflect structural limitations of the health system and broader socioeconomic inequalities. Although administrative data are valuable for analyzing national patterns, they should be complemented by studies incorporating clinical detail, qualitative perspectives, and patient-centered outcomes. A multifaceted and sustained approach is essential to reduce inequities and improve the quality and continuity of oncological care in the country.

## Figures and Tables

**Table 1:** Annual Percent Change (APC) of Malignant Neoplasms of the Colon (ICD-10 C18) and Rectum (ICD-10 C20) by Geographic Region, Age Group, and Sex in Brazil, 2013–2024.

Region	Age Group	Diagnoses of Malignant Neoplasms			
		Men	Women	Men	Women
		Colon	Colon	Rectum	Rectum
North	40–44	8.43 (<0.001)	9.63 (0.004)	11.21 (0.018)	16.4 (0.010)
	45–49	14.18 (<0.001)	15.95 (<0.001)	23.0 (0.033)	25.28 (0.030)
	50–54	20.67 (<0.001)	22.58 (0.001)	25.8 (0.002)	32.48 (0.002)
	55–59	21.47 (<0.001)	23.83 (<0.001)	37.01 (0.040)	32.65 (0.017)
	60–64	24.69 (0.001)	25.24 (<0.001)	37.68 (0.019)	37.02 (0.003)
	65–69	23.22 (<0.001)	23.84 (<0.001)	33.96 (0.010)	34.35 (0.006)
	70–74	21.46 (<0.001)	20.88 (0.004)	29.69 (0.031)	28.24 (0.021)
	75–79	15.21 (0.002)	11.08 (<0.001)	21.57 (0.006)	19.3 (0.014)
	80+	7.25 (0.002)	5.72 (<0.001)	7.81 (0.037)	5.43 (0.008)
Northeast	40–44	11.59 (<0.001)	14.14 (<0.001)	19.25 (<0.001)	25.56 (<0.001)
	45–49	18.98 (<0.001)	20.73 (<0.001)	30.55 (<0.001)	36.71 (<0.001)
	50–54	23.83 (<0.001)	26.79 (<0.001)	38.73 (0.002)	49.33 (0.001)
	55–59	32.68 (<0.001)	32.37 (<0.001)	47.38 (0.002)	53.93 (0.006)
	60–64	36.57 (<0.001)	31.96 (<0.001)	52.66 (0.001)	56.11 (0.001)
	65–69	37.5 (<0.001)	29.25 (<0.001)	53.7 (0.003)	51.08 (0.004)
	70–74	31.29 (<0.001)	26.71 (<0.001)	45.23 (0.010)	39.11 (0.003)
	75–79	25.74 (<0.001)	17.28 (<0.001)	26.31 (0.004)	26.25 (0.003)
	80+	11.06 (<0.001)	8.12 (<0.001)	11.22 (0.049)	8.71 (0.026)
Southeast	40–44	17.89 (<0.001)	17.5 (<0.001)	30.29 (<0.001)	39.33 (0.001)
	45–49	30.64 (<0.001)	29.59 (<0.001)	46.35 (<0.001)	57.7 (<0.001)
	50–54	47.61 (<0.001)	39.24 (<0.001)	71.55 (0.001)	79.92 (0.002)
	55–59	64.29 (<0.001)	48.97 (<0.001)	94.81 (0.001)	98.48 (0.003)
	60–64	72.82 (<0.001)	51.09 (<0.001)	118.18 (0.001)	108.35 (0.004)
	65–69	76.4 (<0.001)	47.4 (<0.001)	112.93 (0.003)	98.7 (0.007)
	70–74	64.45 (<0.001)	36.3 (<0.001)	102.37 (0.003)	74.14 (0.007)
	75–79	44.36 (<0.001)	26.98 (<0.001)	68.84 (0.009)	50.57 (0.008)
	80+	20.69 (<0.001)	11.83 (<0.001)	26.16 (0.021)	17.12 (0.025)
South	40–44	28.88 (<0.001)	27.68 (<0.001)	53.13 (0.007)	67.49 (0.012)
	45–49	46.1 (<0.001)	40.6 (<0.001)	82.63 (0.003)	98.79 (0.005)
	50–54	66.66 (<0.001)	53.67 (<0.001)	116.71 (0.005)	114.65 (0.006)
	55–59	89.63 (<0.001)	58.44 (<0.001)	145.17 (0.005)	134.14 (0.007)
	60–64	97.14 (<0.001)	59.92 (<0.001)	168.73 (0.002)	142.01 (0.007)
	65–69	92.79 (<0.001)	55.33 (<0.001)	183.04 (0.006)	142.57 (0.005)
	70–74	83.7 (<0.001)	49.8 (<0.001)	153.18 (0.010)	115.09 (0.009)
	75–79	58.84 (<0.001)	34.62 (<0.001)	109.44 (0.006)	80.38 (0.014)
	80+	32.15 (<0.001)	16.76 (<0.001)	46.5 (0.014)	30.66 (0.022)
Center-West	40–44	16.07 (<0.001)	15.27 (<0.001)	31.29 (0.001)	38.39 (0.011)
	45–49	26.04 (<0.001)	22.74 (<0.001)	46.41 (<0.001)	60.21 (0.001)
	50–54	35.27 (<0.001)	34.05 (<0.001)	59.85 (<0.001)	71.63 (0.002)
	55–59	48.39 (<0.001)	40.01 (<0.001)	71.71 (0.001)	78.02 (0.004)
	60–64	54.8 (<0.001)	40.11 (<0.001)	86.75 (<0.001)	75.78 (0.003)
	65–69	53.99 (<0.001)	37.12 (<0.001)	84.62 (0.001)	77.79 (0.001)
	70–74	48.92 (<0.001)	34.81 (<0.001)	77.45 (0.004)	59.13 (<0.001)
	75–79	37.14 (<0.001)	20.45 (<0.001)	50.43 (<0.001)	41.69 (<0.001)
	80+	17.3 (<0.001)	10.2 (<0.001)	22.37 (0.049)	15.99 (0.008)

APC: Annual Percent Change. Values represent the annual percent change in age-adjusted rates, estimated using Prais–Winsten regression. Statistically significant trends are indicated by *p* < 0.05. ICD-10 C18: malignant neoplasm of the colon; ICD-10 C20: malignant neoplasm of the rectum.

**Table 2:** Annual Percent Change (APC) of Patients Undergoing Treatment for Malignant Neoplasms of the Colon (ICD-10 C18) and Rectum (ICD-10 C20) by Geographic Region, Age Group, and Sex in Brazil.

Region	Age Group	Treatment of Malignant Neoplasms			
		Men	Women	Men	Women
		Colon	Colon	Rectum	Rectum
North	40–44	9.78 (<0.001)	12.31 (<0.001)	7.45 (<0.001)	7.97 (<0.001)
	45–49	17.00 (0.015)	19.31 (0.008)	13.28 (<0.001)	13.96 (0.002)
	50–54	19.45 (0.001)	23.49 (<0.001)	18.55 (<0.001)	19.34 (<0.001)
	55–59	26.26 (0.037)	23.05 (0.001)	19.46 (<0.001)	21.04 (<0.001)
	60–64	26.77 (0.003)	28.08 (<0.001)	21.37 (0.016)	22.02 (0.001)
	65–69	25.80 (0.001)	25.37 (0.001)	20.90 (<0.001)	20.81 (0.001)
	70–74	20.25 (0.022)	20.26 (0.004)	19.06 (<0.001)	18.03 (0.008)
	75–79	16.25 (0.001)	13.89 (0.002)	13.07 (0.002)	9.15 (<0.001)
	80+	4.26 (0.003)	3.91 (0.001)	5.79 (0.013)	4.66 (<0.001)
Northeast	40–44	16.53 (0.001)	21.51 (0.001)	10.26 (<0.001)	12.25 (<0.001)
	45–49	26.46 (0.001)	30.96 (<0.001)	16.83 (<0.001)	17.43 (<0.001)
	50–54	31.98 (0.001)	40.02 (<0.001)	22.10 (<0.001)	22.21 (<0.001)
	55–59	38.71 (0.001)	44.14 (0.001)	28.66 (<0.001)	25.72 (<0.001)
	60–64	45.21 (0.001)	45.05 (<0.001)	32.21 (<0.001)	26.75 (<0.001)
	65–69	44.06 (0.001)	40.39 (0.001)	32.72 (<0.001)	24.75 (<0.001)
	70–74	36.58 (0.002)	32.33 (<0.001)	26.43 (<0.001)	22.75 (<0.001)
	75–79	20.41 (0.001)	20.98 (<0.001)	22.20 (<0.001)	14.82 (<0.001)
	80+	7.90 (0.011)	5.98 (0.009)	9.37 (<0.001)	6.83 (<0.001)
Southeast	40–44	23.18 (0.001)	29.48 (<0.001)	15.10 (<0.001)	14.10 (<0.001)
	45–49	37.65 (0.001)	44.88 (<0.001)	27.24 (<0.001)	24.56 (<0.001)
	50–54	55.45 (0.001)	60.07 (<0.001)	41.03 (<0.001)	32.40 (<0.001)
	55–59	74.89 (0.001)	72.97 (<0.001)	55.32 (<0.001)	39.75 (<0.001)
	60–64	90.28 (0.001)	77.03 (<0.001)	61.87 (<0.001)	41.17 (<0.001)
	65–69	92.27 (0.001)	70.82 (<0.001)	62.08 (<0.001)	37.95 (<0.001)
	70–74	75.41 (0.001)	54.53 (<0.001)	53.37 (<0.001)	29.09 (<0.001)
	75–79	51.15 (0.001)	36.83 (<0.001)	36.20 (<0.001)	22.25 (<0.001)
	80+	18.05 (0.001)	11.45 (0.003)	16.47 (0.002)	9.69 (<0.001)
South	40–44	41.83 (0.001)	46.21 (<0.001)	25.40 (<0.001)	23.41 (<0.001)
	45–49	63.71 (0.001)	68.89 (<0.001)	40.61 (<0.001)	33.09 (<0.001)
	50–54	87.81 (0.005)	77.85 (<0.001)	57.66 (<0.001)	42.00 (<0.001)
	55–59	109.21 (0.003)	93.38 (<0.001)	76.83 (<0.001)	47.47 (<0.001)
	60–64	124.29 (0.001)	98.99 (<0.001)	83.89 (<0.001)	48.63 (<0.001)
	65–69	138.21 (0.001)	99.87 (<0.001)	77.15 (<0.001)	47.74 (<0.001)
	70–74	107.37 (0.001)	80.90 (<0.001)	69.68 (<0.001)	41.33 (<0.001)
	75–79	79.71 (0.001)	58.16 (0.001)	48.68 (<0.001)	28.50 (<0.001)
	80+	31.36 (0.002)	21.53 (0.002)	25.51 (<0.001)	13.88 (<0.001)
Midwest	40–44	24.09 (0.001)	28.91 (0.001)	14.41 (<0.001)	12.92 (<0.001)
	45–49	38.53 (0.001)	47.98 (<0.001)	24.07 (<0.001)	19.38 (<0.001)
	50–54	50.26 (0.001)	57.56 (<0.001)	32.00 (<0.001)	29.74 (<0.001)
	55–59	60.38 (0.001)	63.43 (<0.001)	43.37 (<0.001)	32.72 (<0.001)
	60–64	71.62 (0.001)	62.53 (<0.001)	47.52 (<0.001)	32.00 (<0.001)
	65–69	73.24 (0.001)	61.95 (0.001)	45.21 (<0.001)	30.23 (<0.001)
	70–74	59.68 (0.001)	47.64 (<0.001)	43.95 (0.003)	29.15 (<0.001)
	75–79	39.55 (0.001)	33.98 (<0.001)	32.17 (<0.001)	17.95 (<0.001)
	80+	15.50 (0.026)	12.69 (0.001)	14.86 (<0.001)	8.54 (<0.001)

**Table 3:** Analysis of rectal cancer (ICD-10 C20) diagnoses by clinical and sociodemographic variables, with risk ratio (RR), according to region of residence, Brazil, 2013–2024.

Characteristics	CIS n (%)	Stage I n (%)	Stage II n (%)	Stage III n (%)	Stage IV n (%)
Sex					
Male	1469 (53.85)	1623 (54.92)	8201 (54.29)	15,513 (53.91)	9312 (56.20)
Female	1259 (46.15)	1332 (45.08)	6906 (45.71)	13,262 (46.09)	7258 (43.80)
RR (Female)	1.17	1.22	1.19	1.17	1.28
Treatment delay					
≤30 days	431 (15.80)	538 (18.21)	2240 (14.83)	3826 (13.30)	3451 (20.83)
31–60 days	618 (22.65)	729 (24.67)	3955 (26.18)	7269 (25.26)	4190 (25.29)
RR (31–60 d)	0.70	0.74	0.57	0.53	0.82
>60 days	1679 (61.55)	1688 (57.12)	8912 (58.99)	17,680 (61.44)	8929 (53.89)
RR (>60 d)	0.26	0.32	0.25	0.22	0.39
Therapeutic modality					
Chemotherapy	1194 (43.77)	1448 (49.00)	8358 (55.33)	16,860 (58.59)	12,812 (77.32)
Radiotherapy	1440 (52.79)	1356 (45.89)	5318 (35.20)	9071 (31.52)	3275 (19.76)
RR (Radio)	0.83	1.07	1.57	1.86	3.91
Both	94 (3.45)	151 (5.11)	1431 (9.47)	2844 (9.88)	483 (2.91)
RR (Both)	12.70	9.59	5.84	5.93	26.53
Region					
North	22 (0.81)	78 (2.64)	655 (4.34)	988 (3.43)	471 (2.84)
Northeast	288 (10.56)	424 (14.35)	2712 (17.95)	5509 (19.15)	2612 (15.76)
RR (NE)	0.08	0.18	0.24	0.18	0.18
Southeast	1760 (64.52)	1400 (47.38)	7550 (49.98)	13,598 (47.26)	7951 (47.98)
RR (SE)	0.01	0.06	0.09	0.07	0.06
South	583 (21.37)	893 (30.22)	3293 (21.80)	6564 (22.81)	4090 (24.68)
RR (South)	0.04	0.09	0.20	0.15	0.12
Center-West	75 (2.75)	160 (5.41)	897 (5.94)	2116 (7.35)	1446 (8.73)
RR (CW)	0.29	0.49	0.73	0.47	0.33

CIS: Carcinoma in situ.

**Table 4:** Analysis of Malignant Neoplasms of the Colon (ICD-10 C18) Clinical Stages by Clinical and Sociodemographic Variables, with risk ratio (RR), according to region of residence, Brazil, 2013–2024.

Characteristics	CIS n (%)	Stage I n (%)	Stage II n (%)	Stage III n (%)	Stage IV n (%)
Sex					
Male	969 (50.6)	820 (47.8)	6430 (49.6)	12,613 (48.9)	17,239 (50.1)
Female	946 (49.4)	894 (52.2)	6542 (50.4)	13,192 (51.1)	17,133 (49.9)
RR (Female)	0.98	1.09	1.02	1.05	0.99
Treatment delay					
≤30 days	439 (22.9)	578 (33.7)	1920 (14.8)	4040 (15.7)	8229 (23.9)
31–60 days	498 (26.0)	440 (25.7)	3829 (29.5)	7926 (30.7)	8793 (25.6)
RR (31–60 d)	1.13	0.76	1.99	1.96	1.07
>60 days	978 (51.1)	696 (40.6)	7223 (55.7)	13,839 (53.6)	17,350 (50.5)
RR (>60 d)	2.23	1.20	3.76	3.42	2.11
Therapeutic modality					
Chemotherapy	1863 (97.3)	1679 (98.0)	12,916 (99.6)	25,719 (99.7)	34,041 (99.0)
Radiotherapy	52 (2.7)	35 (2.0)	56 (0.4)	85 (0.3)	328 (0.9)
RR (Radio)	0.03	0.02	0.00	0.00	0.01
Both	0 (0.0)	0 (0.0)	0 (0.0)	1 (0.0)	3 (0.0)
RR (Both)	0.00	0.00	0.00	0.00	0.00
Region					
North	12 (0.6)	37 (2.2)	362 (2.8)	727 (2.8)	803 (2.3)
Northeast	218 (11.4)	162 (9.4)	2794 (21.5)	4737 (18.4)	5726 (16.7)
RR (NE)	18.06	4.37	7.72	6.51	7.12
Southeast	1208 (63.1)	561 (32.7)	6097 (47.0)	12,712 (49.3)	16,020 (46.6)
RR (SE)	100.13	15.15	16.85	17.47	19.92
South	361 (18.9)	923 (53.9)	2881 (22.2)	6050 (23.4)	9080 (26.4)
RR (South)	29.92	24.93	7.96	8.32	11.29
Center-West	116 (6.1)	31 (1.8)	838 (6.5)	1579 (6.1)	2743 (8.0)
RR (CW)	9.62	0.84	2.32	2.17	3.41

CIS: carcinoma in situ. RR: risk ratio. Clinical staging follows PAINEL-Oncologia (INCA/DATASUS) classifications. RR values compare each category with the reference group within the same variable. Extremely high or zero RRs in some cells reflect sparse data, particularly for the “Both” therapeutic modality, which had very low frequencies.

**Table 5:** Analysis of Malignant Neoplasms of the Colon (ICD-10 C18) Clinical Stages by Treatment, with risk ratio (RR), according to region of residence, Brazil, 2013–2024.

Characteristics	CIS n (%)	Stage I n (%)	Stage II n (%)	Stage III n (%)	Stage IV n (%)
Sex					
Male	1056 (50.5)	953 (47.6)	7259 (49.4)	14,472 (48.9)	19,601 (50.4)
Female	1037 (49.5)	1047 (52.4)	7435 (50.6)	15,105 (51.1)	19,294 (49.6)
RR (Female)	0.98	1.10	1.02	1.04	0.98
Treatment delay					
≤30 days	489 (23.4)	649 (32.5)	2170 (14.8)	4652 (15.7)	9436 (24.3)
31–60 days	553 (26.4)	542 (27.1)	4367 (29.7)	9138 (30.9)	10,091 (25.9)
RR (31–60 d)	1.13	0.84	2.01	1.96	1.07
>60 days	1051 (50.2)	809 (40.5)	8157 (55.5)	15,787 (53.4)	19,368 (49.8)
RR (>60 d)	2.15	1.25	3.76	3.39	2.05
Therapeutic modality					
Chemotherapy	2036 (97.3)	1964 (98.2)	14,633 (99.6)	29,484 (99.7)	38,537 (99.1)
Radiotherapy	57 (2.7)	36 (1.8)	61 (0.4)	92 (0.3)	354 (0.9)
RR (Radio)	0.03	0.02	0.00	0.00	0.01
Region					
North	13 (0.6)	51 (2.5)	362 (2.8)	829 (2.8)	887 (2.3)
Northeast	237 (11.3)	184 (9.2)	2794 (21.5)	5443 (18.4)	6403 (16.5)
RR (NE)	18.26	3.61	7.72	6.57	7.22
Southeast	1313 (62.7)	665 (33.2)	6097 (47.0)	14,620 (49.4)	18,340 (47.1)
RR (SE)	101.18	13.04	16.85	17.65	20.68
South	409 (19.5)	1070 (53.5)	2881 (22.2)	7014 (23.7)	10,449 (26.9)
RR (South)	31.52	20.98	7.96	8.47	11.78
Center-West	121 (5.8)	30 (1.5)	838 (6.5)	1671 (5.7)	2816 (7.2)
RR (CW)	9.32	0.59	2.32	2.02	3.18

CIS: carcinoma in situ; RR: risk ratio. Clinical staging follows PAINEL-Oncologia (INCA/DATASUS) classifications. RR values compare each category with the corresponding reference group within the same variable. Extremely high or zero RRs in some cells reflect sparse data, particularly for the “Both” therapeutic modality, which had very low frequencies.

**Table 6:** Analysis of rectal cancer (ICD-10 C20) clinical stages by treatment, with risk ratio (RR), according to region of residence, Brazil, 2013–2024.

Characteristics	CIS (%)	Stage I n (%)	Stage II n (%)	Stage III n (%)	Stage IV n (%)
Sex					
Male	1606 (54.0)	1818 (55.0)	8955 (54.3)	17,258 (54.0)	4998 (50.7)
Female	1369 (46.0)	1490 (45.0)	7524 (45.7)	14,708 (46.0)	4865 (49.3)
RR (Female)	0.85	0.82	0.84	0.85	0.97
Treatment delay					
≤30 days	496 (16.7)	582 (17.6)	2410 (14.6)	4242 (13.3)	3888 (21.1)
31–60 days	671 (22.6)	810 (24.5)	4306 (26.1)	8098 (25.3)	4715 (25.6)
RR (31–60 d)	1.35	1.39	1.79	1.91	1.21
>60 days	1808 (60.8)	1916 (57.9)	9763 (59.2)	19,626 (61.4)	9812 (53.3)
RR (>60 d)	3.65	3.29	4.05	4.63	2.52
Therapeutic modality					
Chemotherapy	1296 (45.1)	1644 (52.3)	9281 (62.1)	18,991 (65.7)	14,315 (80.1)
Radiotherapy	1577 (54.9)	1497 (47.7)	5675 (37.9)	9898 (34.3)	3546 (19.9)
RR (Radio)	1.22	0.91	0.61	0.52	0.25
Region					
North	20 (0.7)	80 (2.4)	685 (4.2)	1068 (3.3)	500 (2.7)
Northeast	312 (10.5)	473 (14.3)	2973 (18.0)	6115 (19.1)	2863 (15.5)
RR (NE)	15.60	5.91	4.34	5.73	5.73
Southeast	1913 (64.3)	1639 (49.5)	8360 (50.7)	15,255 (47.7)	9070 (49.3)
RR (SE)	95.65	20.49	12.20	14.28	18.14
South	652 (21.9)	976 (29.5)	3599 (21.8)	7339 (23.0)	4544 (24.7)
RR (South)	32.60	12.20	5.25	6.87	9.09
Center-West	78 (2.6)	140 (4.2)	862 (5.2)	2189 (6.8)	1438 (7.8)
RR (CW)	3.90	1.75	1.26	2.05	2.88

CIS: carcinoma in situ; RR: risk ratio. Clinical staging and treatment classifications follow PAINEL-Oncologia (INCA/DATASUS). RR values compare each category with the corresponding reference group within the same variable. Extremely high or low RR values in some cells reflect sparse data or low-frequency categories, particularly for regional strata and treatment modalities.

## Data Availability

Data supporting the findings of this study are publicly available at the Brazilian Ministry of Health’s PAINEL-Oncologia platform (https://paineis.saude.gov.br/paineis/oncologia/, accessed on 11 June 2025). No additional datasets were generated or analyzed beyond these publicly accessible sources.
